# Advancements in Nanoporous Materials for Biomedical Imaging and Diagnostics

**DOI:** 10.3390/jfb15080226

**Published:** 2024-08-14

**Authors:** Nargish Parvin, Vineet Kumar, Tapas Kumar Mandal, Sang Woo Joo

**Affiliations:** School of Mechanical Engineering, Yeungnam University, Gyeongsan 38541, Republic of Korea; nargish.parvin@gmail.com (N.P.); vineetfri@gmail.com (V.K.)

**Keywords:** nanoporous materials, biomedical imaging, diagnostics, functionalization techniques, multimodal imaging

## Abstract

This review explores the latest advancements in nanoporous materials and their applications in biomedical imaging and diagnostics. Nanoporous materials possess unique structural features, including high surface area, tunable pore size, and versatile surface chemistry, making them highly promising platforms for a range of biomedical applications. This review begins by providing an overview of the various types of nanoporous materials, including mesoporous silica nanoparticles, metal–organic frameworks, carbon-based materials, and nanoporous gold. The synthesis method for each material, their current research trends, and prospects are discussed in detail. Furthermore, this review delves into the functionalization and surface modification techniques employed to tailor nanoporous materials for specific biomedical imaging applications. This section covers chemical functionalization, bioconjugation strategies, and surface coating and encapsulation methods. Additionally, this review examines the diverse biomedical imaging techniques enabled by nanoporous materials, such as fluorescence imaging, magnetic resonance imaging (MRI), computed tomography (CT) imaging, ultrasound imaging, and multimodal imaging. The mechanisms underlying these imaging techniques, their diagnostic applications, and their efficacy in clinical settings are thoroughly explored. Through an extensive analysis of recent research findings and emerging trends, this review underscores the transformative potential of nanoporous materials in advancing biomedical imaging and diagnostics. The integration of interdisciplinary approaches, innovative synthesis techniques, and functionalization strategies offers promising avenues for the development of next-generation imaging agents and diagnostic tools with enhanced sensitivity, specificity, and biocompatibility.

## 1. Introduction

### 1.1. Background and Importance

Nanoporous materials have garnered significant attention in the field of biomedical research because of their special qualities, which include high surface area, tunable pore sizes, and exceptional chemical and thermal stability. These materials have shown tremendous potential for enhancing several applications in biomedicine, particularly in imaging and diagnostics, where precision and specificity are paramount. The capability to precisely control their physical and chemical properties enables nanoporous materials to engage in extremely specialized interactions with biological systems, thereby improving the efficacy and accuracy of biomedical imaging and diagnostic techniques.

Mesoporous silica nanoparticles (MSNs) are among the most widely studied nanoporous materials. MSNs have demonstrated considerable promise due to their biocompatibility, ease of functionalization, and high loading capacity for imaging agents [1,2,3]. Similarly, metal–organic frameworks (MOFs) offer an adaptable platform for biomedical applications due to their structural diversity and ability to incorporate a wide range of functional groups [4,5]. MOFs have been explored for their potential to enhance imaging contrast and enable targeted delivery of diagnostic agents [4,6]. Additionally, porous silicon nanoparticles are being developed for their biodegradability and ability to improve the resolution of imaging techniques [7,8].

Carbon-based nanoporous materials, such as carbon nanotubes and graphene oxide, have also been widely researched for their potential in bioimaging applications due to their unique electrical and optical properties [9]. Moreover, nanoporous gold has shown promise for biosensing and imaging due to its high surface area and plasmonic properties [10]. The integration of these materials into various imaging techniques, such as fluorescence imaging, magnetic resonance imaging (MRI), and computed tomography (CT), has led to significant advancements in the field [7,11,12].

### 1.2. Scope and Objectives

The goal of this review is to give a thorough summary of current developments in nanoporous materials for biomedical imaging and diagnostics. The scope of this review includes a thorough discussion of various types of nanoporous materials, their synthesis techniques, and functionalization methods. Additionally, the review will investigate the employment of these materials in different imaging techniques and their role in disease diagnostics.

The objectives of this review are as follows:

1. Summarize the types and properties of nanoporous materials utilized in biomedical applications.
2. Discuss state-of-the-art synthesis and functionalization techniques that enhance the performance of nanoporous materials.
3. Evaluate the use of nanoporous materials in various imaging and diagnostic applications, highlighting their mechanisms and efficacy.
4. Identify the challenges and limitations associated with the use of nanoporous materials in biomedical contexts.
5. Provide insights into future perspectives and potential breakthroughs in the field.

**Figure 1:**
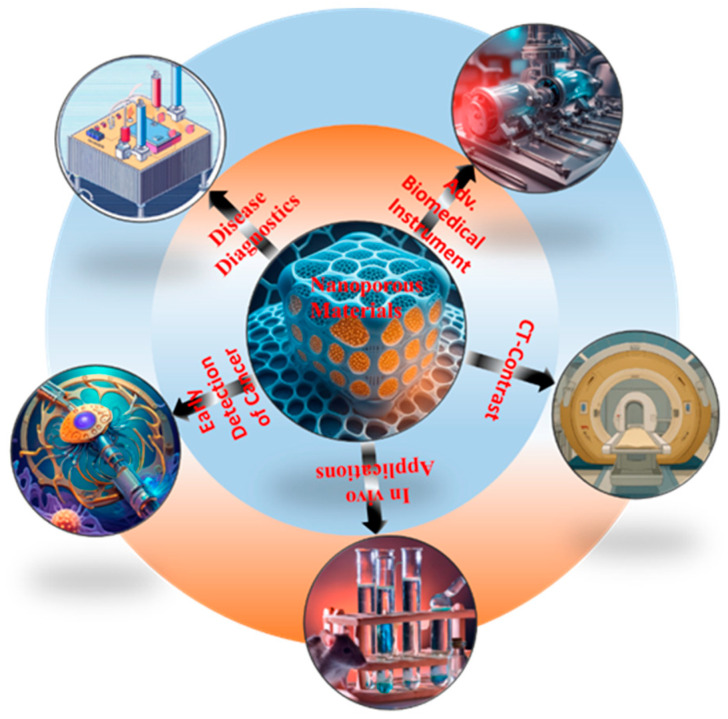
Schematic diagram of porous nanomaterials biomedical applications.

By consolidating current knowledge and highlighting future research directions, the purpose of this review is to be a useful stock for researchers and practitioners working in the domain of biomedical imaging and diagnostics [13,14,15]. Figure 1 provides a schematic of porous nanomaterials and their biomedical applications, highlighting their unique structural features, including high surface area, tunable pore size, and functionalizable surfaces. These properties enable their use in various biomedical applications, such as targeted drug delivery, where the pores can encapsulate therapeutic agents, and in imaging, where they enhance contrast. The figure also depicts the potential of these materials in biosensing and tissue engineering, demonstrating their versatility in the medical field.

## 2. Types of Nanoporous Materials

Nanoporous materials encompass a diverse array of structures, including mesoporous silica nanoparticles (MSNs), metal–organic frameworks (MOFs), carbon-based materials, and nanoporous metals. Current research is focused on optimizing synthesis methods and functionalization techniques to tailor their properties for specific biomedical applications. Prospects include enhancing drug delivery efficiency, improving imaging contrast, and enabling targeted therapy. As researchers delve deeper into understanding the structure–property relationships of nanoporous materials, the potential for breakthroughs in precision medicine and personalized healthcare continues to grow, promising novel solutions for diagnosing and treating diseases at the molecular level.

### 2.1. Mesoporous Silica Nanoparticles

Mesoporous silica nanoparticles (MSNs) have become a focal point in current biomedical research because of their superior biocompatibility, high surface area, and versatile functionalization capabilities. These properties make MSNs ideal for drug delivery, bioimaging, and diagnostic applications. Recent research has shown the efficacy of MSNs in enhancing the delivery and controlled release of therapeutic agents, thereby improving treatment outcomes [3]. Additionally, MSNs have been employed as carriers for imaging agents, significantly enhancing contrast in various imaging techniques, including fluorescence and MRI [16,17]. Looking forward, researchers aim to further optimize MSN surface modification techniques to improve target specificity and reduce potential cytotoxicity, thereby expanding their utility in precision medicine [13].

### 2.2. Metal–Organic Frameworks (MOFs)

**Figure 2:**
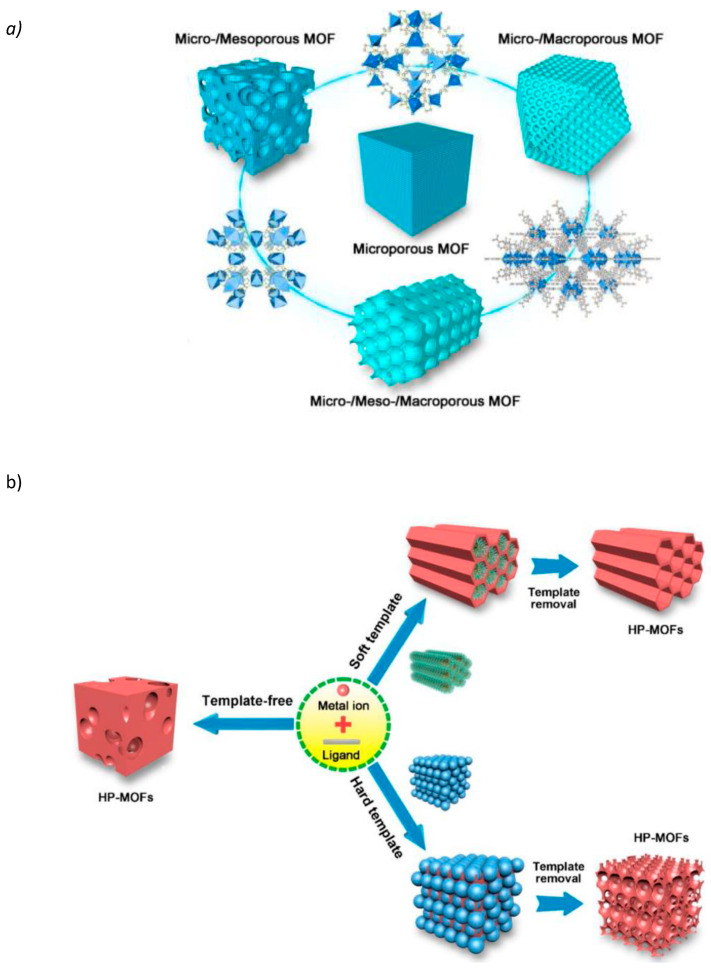
(**a**) Schematic diagram of HP-MOFs. (**b**) Diagrammatic overview of the synthesis methods used to produce HP-MOFs. Reprinted with permission from Ref. [22] https://doi.org/10.1016/j.jhazmat.2020.122765.

Metal–organic frameworks (MOFs) have emerged as promising candidates for various biomedical applications due to their tunable pore size, high surface area, and customizable functionalities. Current research focuses on hierarchical porous metal–organic frameworks (HP-MOFs) for drug delivery, imaging, and sensing applications [18]. The unique structural properties of MOFs allow for the encapsulation and controlled release of medicinal substances, improving the effectiveness of treatment [19]. Additionally, MOFs have demonstrated potential as contrast agents in multiple imaging techniques, enabling accurate disease diagnosis and monitoring [20]. Future research aims to optimize MOF synthesis techniques and explore their potential in specific drug administration and theranostic uses [20,21]. Figure 2 presents a schematic of high-performance metal–organic frameworks (HP-MOFs) and their synthesis techniques. The figure illustrates the various stages involved in creating HP-MOFs, including the selection of organic linkers and metal ions, which are crucial for determining the framework’s properties. It also highlights the different synthesis methods, such as solvothermal, hydrothermal, and microwave-assisted techniques, used to construct these frameworks. Additionally, the figure showcases the structural diversity and unique features of HP-MOFs, such as their porosity and surface area, which are essential for their applications in gas storage, catalysis, and drug delivery [22].

### 2.3. Carbon-Based Nanoporous Materials

Carbon-based nanoporous materials, such as carbon nanotubes and graphene oxide, are currently the focus of extensive research in biomedical applications due to their unique electrical and optical properties. Recent studies have explored their potential in drug delivery, biosensing, and bioimaging applications [23]. These materials are excellent choices for targeted drug administration because of their high surface area, biocompatibility, simplicity of functionalization, and imaging contrast enhancement [24]. Future research aims to further optimize their properties and explore novel applications in personalized medicine and theranostics [25].

### 2.4. Nanoporous Gold

Nanoporous gold (NPG) has recently come to light as a potential material for biomedical uses because of its distinctive structural and optical characteristics. Current research focuses on utilizing NPG for biosensing, drug delivery, and imaging applications [26]. The high surface area and tunable pore size of NPG permit effective biomolecule immobilization and enhanced sensitivity in biosensing platforms [27]. Moreover, NPG’s plasmonic properties make it an attractive candidate for enhancing imaging contrast and enabling targeted therapy [28]. Future prospects include exploring NPG’s potential in theranostics and personalized medicine applications.

## 3. Synthesis Techniques

Advancements in the synthesis techniques of porous materials are pivotal in tailoring their properties for biomedical applications. Current research focuses on developing innovative techniques like sol–gel, template-directed, and self-assembly methods to control pore size, surface chemistry, and morphology. These techniques enable precise engineering of porous materials, optimizing their performance in drug delivery, imaging, and sensing applications. Prospects include the integration of additive manufacturing and bio-inspired synthesis methods to create complex, hierarchical structures with enhanced functionalities. By pushing the boundaries of synthesis techniques, researchers aim to realize the complete efficiency of porous materials in revolutionizing biomedical technologies for improved diagnosis and treatment strategies.

### 3.1. Sol–Gel Method

**Figure 3:**
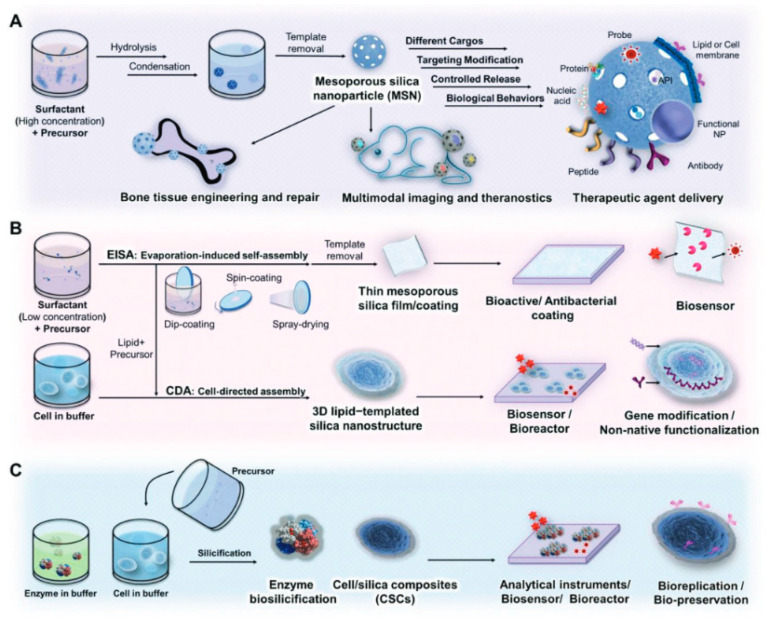
Schematic of advanced porous silica materials based on the sol–gel method in biomedical uses. (**A**) Mesoporous silica nanoparticles (MSNs). (**B**) Silica films with nanostructures synthesized by evaporation-induced self-assembly. (**C**) Silicification through biomimicry. Reprinted with permission from Ref. [30] https://doi.org/10.1002/adfm.201909539.

The sol–gel method is a versatile synthesis technique widely used for porous nanomaterial fabrication and crucial for various biomedical applications. Through controlled hydrolysis and condensation of precursor molecules, this method allows precise tailoring of pore size, surface area, and morphology. Recent studies have highlighted its efficacy in producing porous nanomaterials with enhanced drug loading and release capabilities [29]. Moreover, sol–gel-derived nanomaterials exhibit excellent biocompatibility, making them promising candidates for drug delivery systems and tissue engineering scaffolds [30]. Figure 3 provides a schematic overview of advanced porous silica materials synthesized via sol–gel methods and their biomedical applications. The figure likely includes mesoporous silica nanoparticles (MSNs), characterized by their uniform pore size and high surface area, making them ideal for drug delivery and imaging applications. Figure 3B shows silica films with nanostructures created through evaporation-induced self-assembly, which can be used in biosensing and diagnostics due to their tailored surface properties. Figure 3C illustrates biomimetic silicification, a process inspired by natural silica formation, offering potential for creating biocompatible materials for tissue engineering and regenerative medicine. Overall, the figure emphasizes the versatility of these advanced silica materials in various biomedical fields [30]. Ongoing research aims to refine sol–gel protocols for optimizing nanomaterial properties and functionalities [31].

### 3.2. Hydrothermal Synthesis

Hydrothermal synthesis is a frequently used method for fabricating porous nanomaterials with exact morphological control, size, and pore structure. This method includes the reaction of precursor components in an aqueous solution under high pressure and temperature. The hydrothermal environment facilitates nucleation, growth, and self-assembly processes, leading to the formation of porous structures with tailored properties. Recent research has demonstrated the versatility of hydrothermal synthesis in producing various nanomaterials, including metal oxides, carbon-based materials, and metal–organic frameworks, for drug delivery, catalysis, and environmental remediation [32,33,34]. Ongoing efforts focus on optimizing reaction parameters and exploring novel precursor materials to further enhance the versatility and efficiency of hydrothermal synthesis in porous nanomaterial fabrication.

### 3.3. Template-Assisted Methods

Template-assisted methods have emerged as the cornerstone in the fabrication of porous nanomaterials, particularly due to their ability to offer precise control over structural parameters such as pore size, morphology, and surface characteristics. These methods rely on the use of templates, which are sacrificial or removable structures that guide the formation of pores within the material during synthesis. The versatility and precision of these methods have made them particularly valuable in various applications, including catalysis, drug delivery, energy storage, and increasingly, in the field of disease diagnosis [35,36,37].

#### 3.3.1. Approach and Mechanisms

The core principle behind template-assisted synthesis is the use of a template that defines the architecture of the resulting porous material. The templates can be broadly categorized into three types, namely: soft templates, hard templates, and sacrificial templates.

1. Soft templates: These include surfactants, block copolymers, and other molecular assemblies that form micelles or liquid crystalline phases. During the synthesis, the precursor material deposits around these micellar structures, and subsequent removal of the template (often through calcination or solvent extraction) leaves behind a porous network. This method is particularly advantageous for creating mesoporous structures with highly uniform pore sizes. However, the removal process can sometimes lead to partial collapse of the structure, affecting the uniformity and integrity of the pores.
2. Hard templates: Colloidal particles (such as silica or polystyrene beads) and other solid materials are used as hard templates. The precursor material coats these hard templates, and upon removal (via etching or dissolution), a highly ordered porous structure is obtained. This method allows for excellent control over the pore size and distribution, but the process can be more complex and costly compared to soft templates.
3. Sacrificial templates: These involve templates that can be selectively removed without affecting the underlying structure. Examples include polymer scaffolds that are thermally decomposable. This approach is highly beneficial for creating hierarchical structures, which are increasingly important in applications requiring multi-functional surfaces, such as biosensing and catalysis.

#### 3.3.2. Advantages of Template-Assisted Methods

The precision and control offered by template-assisted methods provide significant advantages, particularly in the context of disease diagnosis. These methods enable the fabrication of nanomaterials with specific pore structures and surface chemistries, which can be tailored to enhance the sensitivity and specificity of diagnostic assays. For example:

- Enhanced sensitivity: The high surface area-to-volume ratio and tunable pore sizes can significantly improve the capture and detection of biomolecules, such as proteins and nucleic acids, which are crucial in diagnostic applications. The uniform pore structure ensures consistent interactions with target molecules, leading to more reliable and reproducible results [35].
- Versatility: The ability to use a wide range of templates allows for the design of materials with diverse properties suitable for various diagnostic techniques, including optical, electrochemical, and magnetic biosensors. This versatility is particularly useful in developing multifunctional diagnostic platforms capable of detecting multiple biomarkers simultaneously [36].
- Scalability: Template-assisted methods can be scaled up for mass production, which is essential for the commercial development of diagnostic tools. The reproducibility of these methods ensures consistent quality across different batches, which is critical for clinical applications where standardization is a key requirement [37].

#### 3.3.3. Disadvantages and Challenges

Despite their advantages, template-assisted methods also present several challenges, including the following:

a. Template Removal: The removal of templates, especially hard templates, can sometimes leave residues that contaminate the porous material, potentially affecting its performance. In biosensing applications, such impurities can lead to false positives or reduce the specificity of the assay.
b. Complexity and cost: The use of hard templates often requires additional steps and specialized equipment for removal, increasing the complexity and cost of the fabrication process. This can be a barrier to the widespread adoption of these methods, particularly in resource-limited settings where low-cost diagnostic tools are needed.
c. Limited scalability for certain templates: While soft templates are relatively easy to scale up, hard templates and some sacrificial templates present challenges in large-scale production. The uniformity and reproducibility of the porous structures can diminish when scaling up, potentially affecting the performance of the resulting diagnostic devices [35].

#### 3.3.4. Applications in Disease Diagnosis

The precision and versatility of template-assisted methods have been leveraged to develop advanced diagnostic tools. For instance, mesoporous silica nanoparticles (MSNs) synthesized using soft template methods have been used for the detection of cancer biomarkers. These nanoparticles can be functionalized with specific ligands or antibodies that selectively bind to cancer-related proteins, allowing for an early and accurate diagnosis [36].

In another example, hierarchical porous carbon materials, synthesized using sacrificial templates, have been employed in electrochemical biosensors for the detection of glucose and cholesterol. The hierarchical structure enhances the electron transfer rate and increases the surface area available for enzyme immobilization, thereby improving the sensitivity and response time of the sensor [37].

#### 3.3.5. Future Directions

Future research in template-assisted methods aims to explore novel templates and synthesis strategies to further enhance the capabilities of nanoporous materials in disease diagnosis. Innovations such as biodegradable templates, which can be removed without harsh chemicals, are being investigated to reduce environmental impact and improve the biocompatibility of the materials [35,36,37].

Moreover, integrating these materials into lab-on-a-chip devices and point-of-care diagnostic platforms holds great promise for personalized medicine. The development of portable, low-cost diagnostic tools that can provide rapid and accurate results at the patient’s bedside is a key goal in this field.

So, while template-assisted methods offer numerous advantages in the fabrication of porous nanomaterials, including precise control over structural features and versatility in application, challenges such as template removal and cost must be addressed. Advances in this field continue to expand the scope and potential of these materials, particularly in the critical area of disease diagnosis. As research progresses, these methods are expected to play an increasingly important role in developing innovative diagnostic technologies that can meet the growing demands of modern healthcare.

### 3.4. Self-Assembly Techniques

Self-assembly techniques are pivotal in the fabrication of porous nanomaterials, offering precise control over structure and morphology through the spontaneous organization of building blocks. This method relies on the natural relationships that exist between molecules or nanoparticles to drive the formation of ordered structures at the nanoscale. Examples include block copolymer self-assembly, DNA origami, and colloidal crystal templating, each offering unique advantages in porous material synthesis [38,39,40]. These techniques enable the creation of porous architectures with tailored pore sizes, surface functionalities, and hierarchical structures, making them extremely appealing for use in drug delivery applications, catalysis, and sensing. Ongoing research focuses on expanding the versatility and scalability of self-assembly methods, as well as exploring new strategies for controlling assembly dynamics and enhancing material properties for advanced nanotechnology applications.

## 4. Functionalization and Surface Modification

### 4.1. Chemical Functionalization

**Figure 4:**
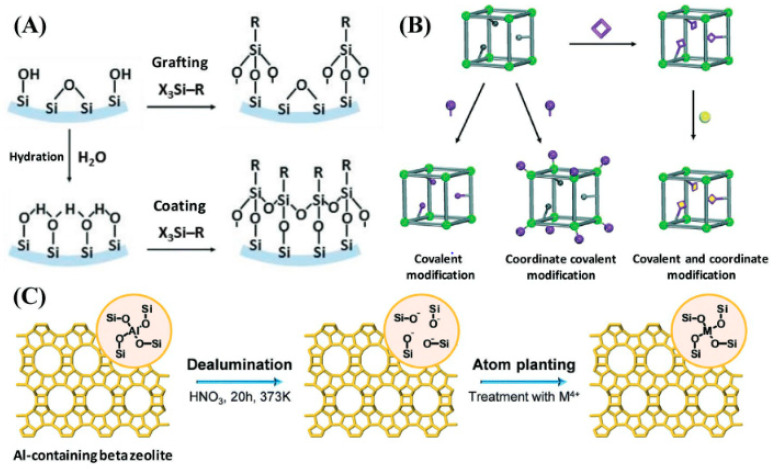
Examples of chemical alterations in porous materials. (**A**) Silica surface chemical functionalization through coating and grafting techniques. (**B**) Various postsynthetic techniques for MOF chemical functionalization. (**C**) Two-step postsynthetic method for the synthesis of zeolites containing metal. Reproduced with permission from Ref. [44] Copyright 2015, Royal Society of Chemistry. https://doi.org/10.1002/adfm.201908371.

Chemical functionalization plays a vital role in customizing the surface characteristics of porous materials for particular applications. Through the covalent attachment of functional groups, such as amino, carboxyl, or hydroxyl moieties, onto the surface of porous materials, their physicochemical properties can be finely tuned. Recent research has demonstrated the versatility of chemical functionalization in enhancing the biocompatibility, stability, and functionality of porous materials for drug delivery, biosensing, and tissue engineering [41,42,43]. Figure 4 illustrates various chemical modifications of porous materials to enhance their functionality and range of applications. Figure 4A shows the functionalization of silica surfaces via coating and grafting techniques, which can modify the surface chemistry for specific applications, such as increasing hydrophilicity or adding reactive groups for further modification. Figure 4B depicts postsynthetic techniques used to chemically functionalize metal–organic frameworks (MOFs), which might include processes like ligand exchange, metal ion incorporation, or covalent attachment of functional groups, allowing for tailored adsorption properties or catalytic activity. Figure 4C shows a two-step postsynthetic method for synthesizing metal-containing zeolites, highlighting the insertion of metal ions into the zeolite framework to enhance catalytic or ion-exchange properties. Overall, the figure emphasizes how these chemical alterations enable the customization of porous materials for specific applications in areas like catalysis, adsorption, and sensing [44]. By carefully selecting functionalization strategies and precursor molecules, researchers can impart desirable properties to porous materials, enabling exact control over how they interact with biological systems and facilitating targeted therapeutic interventions.

### 4.2. Bioconjugation Strategies

Bioconjugation strategies enable the coupling of biomolecules, like proteins, antibodies, or nucleic acids, onto the surface of porous materials, imparting them with biological functionality. This process facilitates the development of biomimetic platforms for targeted drug delivery, biosensing, and biomedical imaging [45,46,47]. Current developments in bioconjugation techniques have resulted in the creation of multifunctional porous materials capable of specific molecular recognition, cellular targeting, and therapeutic payload delivery. By harnessing the specificity and versatility of bioconjugation reactions, researchers aim to engineer next-generation porous materials with enhanced biocompatibility and tailored functionality for various biomedical applications.

### 4.3. Surface Coating and Encapsulation

Surface coating and encapsulation techniques are pivotal for enhancing the surface properties and functional capabilities of porous materials. These modifications can significantly improve the stability, biocompatibility, and overall functionality of the materials, making them more suitable for biomedical applications. For instance, applying biocompatible polymers such as polyethylene glycol (PEG), poly(lactic-co-glycolic acid) (PLGA), and chitosan can enhance the stability and reduce the immunogenicity of the porous structures [48,49,50]. PEGylation, in particular, is widely used to prolong the circulation time of nanoparticles in the bloodstream by preventing opsonization and subsequent clearance by the reticuloendothelial system (RES).

Additionally, lipid bilayer coatings, which mimic cell membranes, provide a natural interface that can facilitate cellular uptake and protect the encapsulated payloads from degradation. Inorganic coatings, such as silica or calcium phosphate, can be used to enhance the mechanical strength and control the degradation rates of porous materials, thereby ensuring a sustained release of drugs or other therapeutic agents. Recent advancements have shown that these surface modifications not only protect the payloads but also allow for targeted delivery by functionalizing the surface with ligands or antibodies. For example, PLGA is often chosen for its biodegradability and ability to deliver a range of therapeutic agents, from small molecules to proteins and nucleic acids. Chitosan, on the other hand, is favored for its mucoadhesive properties and is often used in drug delivery systems targeting mucosal tissues [50].

Continued research in this field is focused on refining these coating techniques and exploring new encapsulation strategies, such as layer-by-layer (LbL) assembly, to create multi-functional porous materials with enhanced biomedical utility. This includes the development of smart coatings that respond to specific biological stimuli, such as pH or temperature changes, to release their payloads in a controlled manner. The use of advanced polymers, such as poly(ethylene oxide)-block-poly(ε-caprolactone) (PEO-b-PCL) and dendrimers, is also being investigated for their potential to improve the encapsulation efficiency and release profiles of various therapeutic agents [49,50].

## 5. Biomedical Imaging Applications

### 5.1. Fluorescence Imaging

Fluorescence imaging is a potential tool in biomedical research and diagnostics, offering high sensitivity and spatial resolution. Porous nanomaterials, such as mesoporous silica nanoparticles (MSNs) and carbon-based nanomaterials, have appeared as powerful contrast agents for fluorescence imaging because of their tunable pore structures and surface functionalization capabilities [51,52,53]. By encsapsulating fluorescent dyes or quantum dots within the pores or modifying the surface with targeting ligands, porous nanoparticles have the ability to concentrate in particular tissues or cells, allowing for accurate in vivo imaging of disease locations.

### 5.2. Magnetic Resonance Imaging (MRI)

Magnetic resonance imaging (MRI) is a non-invasive imaging technique that provides high-resolution anatomical and functional information. Porous nanomaterials, such as iron oxide nanoparticles and metal–organic frameworks (MOFs), have attracted significant interest as contrast agents for MRI as they have tunable magnetic properties and biocompatibility [54,55,56]. These nanomaterials can be made functional with targeting ligands or encapsulated with imaging agents to increase their sensitivity and specificity for detecting diseases such as cancer and cardiovascular disorders.

### 5.3. Computed Tomography (CT) Imaging

**Figure 5:**
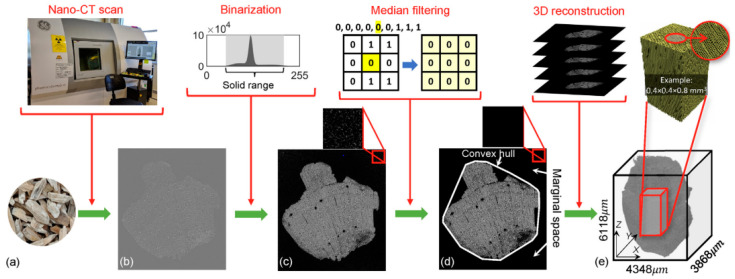
Loblolly pine 3D microstructural topology reconstruction workflow based on nano-CT: (**a**) particles of loblolly pine; (**b**) slice of raw CT image; (**c**) slice of binarized; (**d**) pre-processed, convex-hulled slice; and (**e**) A three-dimensional digitally recreated particle. The approaches utilized for each stage are highlighted in the top row. Reprinted with permission from Ref. [60] https://doi.org/10.1016/j.powtec.2021.05.006.

Computed tomography (CT) imaging is a broadly applied diagnostic tool that offers thorough cross-sectional images of the body. Porous nanomaterials, such as gold nanoparticles and iodine-based nanoparticles, have gained attention as contrast agents for CT imaging as they have high X-ray attenuation coefficiensts and biocompatibility [57,58,59]. Figure 5 outlines the 3D microstructural topology reconstruction workflow for loblolly pine using nano-CT imaging. The process begins with the physical particles of loblolly pine (Figure 5a). These particles are scanned, producing a raw CT image slice (Figure 5b). In Figure 5c, this slice is binarized, distinguishing between the solid material and voids within the sample. The binarized image is then pre-processed and refined using convex hulling techniques to enhance the accuracy of the structure’s representation (Figure 5d). Finally, Figure 5e presents a three-dimensional digital reconstruction of the particle, allowing for detailed analysis of its microstructure. This figure highlights the step-by-step process and methodologies applied at each stage to achieve a precise 3D model of the pine’s internal topology [60].

### 5.4. Ultrasound Imaging

Ultrasound imaging is a widely used medical imaging technique that employs high-frequency sound waves to visualize internal body structures in real time. Porous nanomaterials, such as gas-filled microbubbles and lipid-based nanoparticles, have emerged as contrast agents for ultrasound imaging as thesy are able to enhance acoustic signals and improve imaging contrast [61,62,63].

### 5.5. Multimodal Imaging

Multimodal imaging, integrating various imaging modalities, offers synergistic advantages for comprehensive biomedical diagnostics and therapeutics. Porous nanomaterials serve as a flexible structure for multimodal imaging because of their adjustable qualities and facile surface functionalization. By involving different imaging agents or contrast agents within their porous structures, these nanomaterials enable simultaneous visualization using multiple imaging methods, like fluorescence, magnetic resonance imaging (MRI), computed tomography (CT), and ultrasound. This multimodal approach enhances sensitivity, resolution, and specificity while providing complementary information for accurate diagnosis and monitoring of diseases [64,65,66]. Additionally, the capacity to tailor the physicochemical characteristics of porous nanomaterials further expands their utility in multimodal imaging, paving the way for advanced biomedical applications.

## 6. Diagnostic Applications

### 6.1. Biosensing and Bioimaging

Porous nanomaterials are revolutionizing biosensing and bioimaging applications by offering enhanced sensitivity, selectivity, and biocompatibility. Functionalized porous materials, like mesoporous silica nanoparticles (MSNs) and carbon-based nanomaterials, serve as excellent platforms for immobilizing biomolecules and detecting target analytes with high specificity [67,68,69]. These materials enable the development of portable biosensors that are affordable for point-of-care diagnostics and real-time monitoring of biomolecular interactions. Moreover, their porous structures can accommodate imaging agents for fluorescent, magnetic resonance, or photoacoustic imaging, facilitating the molecular-level observation of biological processes without intrusive methods.

### 6.2. Disease Diagnostics

Porous nanomaterials offer promising prospects in disease diagnostics, enabling rapid and accurate detection of biomarkers associated with various diseases. Functionalized porous materials, like metal–organic frameworks (MOFs) and nanoporous polymers, can selectively capture target molecules from complex biological samples, facilitating sensitive detection using techniques like surface-enhanced Raman scattering (SERS) or electrochemical sensing [70,71,72]. These materials hold great potential for the early diagnosis of infectious diseases, cancer, and neurological disorders, offering improved sensitivity, specificity, and multiplexing capabilities compared to csonventional diagnostic methods.

### 6.3. Early Detection of Cancer

**Figure 6:**
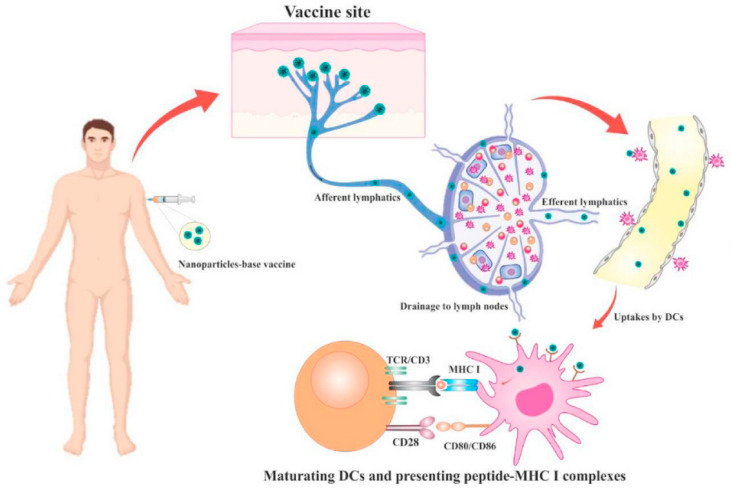
Schematic illustration of the lymph node-targeting NPs-induced adaptive immune response driven by antigen. Reprinted with permission from Ref. [74] https://doi.org/10.1016/j.mtchem.2022.101144.

Porous nanomaterials hold immense potential for the early detection of cancer, offering sensitive and specific detection of cancer biomarkers in biological fluids and tissues. Functionalized porous materials, such as nanoporous gold and dendritic mesoporous silica nanoparticles, can selectively capture circulating tumor cells or tumor-derived exosomes for liquid biopsy-based cancer diagnostics [73,74,75]. Figure 6 provides a schematic overview of how lymph node-targeting nanoparticles (NPs) induce an adaptive immune response through antigen delivery. The diagram illustrates nanoparticles designed to specifically target lymph nodes, which are crucial for initiating immune responses. Once inside the lymph nodes, these NPs release antigens that are recognized by antigen-presenting cells (APCs). The APCs then process and present the antigens to T cells, activating them. This activation leads to a robust adaptive immune response, characterized by the production of antigen-specific T and B cells. The figure emphasizes the strategic role of NPs in enhancing immune response efficiency and specificity [74]. These materials enable non-invasive and real-time monitoring of cancer progression, facilitating early intervention and personalized treatment strategies. Furthermore, their compatibility with various imaging techniques allows for multimodal imaging of tumors, offering comprehensive information for accurate diagnosis and therapeutic monitoring.

### 6.4. In Vivo and In Vitro Applications

Porous nanomaterials find diverse applications in both in vivo and in vitro diagnostic techniques, offering versatile platforms for biomolecular detection, imaging, and therapeutic delivery. These materials can be functionalized in vivo with targeting ligands or imaging agents for real-time monitoring of disease progression and treatment response [76,77,78]. In addition, porous nanomaterials serve as sensitive and selective biosensing platforms in vitro for point-of-care diagnostics and high-throughput screening of biomarkers [79,80,81]. Their biocompatibility, tunable properties, and multifunctionality make them indispensable tools for advancing precision medicine and improving patient outcomes.

## 7. Mechanisms and Efficacy

### 7.1. Imaging Mechanisms

Porous nanomaterials can be used in a variety of imaging mechanisms that enable the precise visualization of biological structures and processes. For example, mesoporous silica nanoparticles (MSNs) can encapsulate fluorescent dyes or quantum dots within their pores, emitting strong fluorescence signals upon excitation, which facilitates fluorescence imaging with high sensitivity and resolution [82,83,84]. Similarly, metal–organic frameworks (MOFs) possess tunable pore sizes and surface functionalities, allowing for the encapsulation of imaging agents or contrast agents for magnetic resonance imaging (MRI) [85]. Additionally, nanoporous gold nanoparticles exhibit strong X-ray attenuation coefficients, making them excellent contrast agents for computed tomography (CT) imaging [86]. Nanoporous iron oxide nanoparticles (IONPs) have shown superior performance in micro-CT and MRI imaging compared to solid or non-hollow superparamagnetic iron oxide nanoparticles (SPIONs). The unique structure of nanoporous IONPs enhances their surface area-to-volume ratio, facilitating greater adsorption of contrast agents and providing more binding sites for functionalization. This increased surface area enables higher payload capacity for MRI contrast agents, resulting in more pronounced signal enhancement. Additionally, their porous nature allows for better water diffusion, improving the efficiency of magnetic resonance relaxation processes, specifically transverse (T2) relaxation [83,84]. Consequently, nanoporous IONPs can produce stronger T2 contrast on MRI scans compared to non-hollow SPIONs. Furthermore, the structural voids within these nanoparticles contribute to decreased particle density, which can enhance contrast in both MRI and micro-CT by amplifying the magnetic susceptibility effects and reducing attenuation, respectively. These features collectively enhance the imaging sensitivity and resolution, making nanoporous IONPs a valuable tool in biomedical imaging.

### 7.2. Diagnostic Mechanisms

In diagnostic applications, porous nanomaterials leverage various mechanisms to detect specific biomarkers or molecular targets associated with diseases. For instance, nanoporous polymers can selectively adsorb target molecules from complex biological samples, enabling sensitive detection by employing surface-enhanced Raman scattering (SERS) or electrochemical sensing techniques [70,87]. Furthermore, functionalized mesoporous silica nanoparticles (MSNs) can be designed to detect and attach to disease-specific targets, facilitating precise molecular imaging and diagnostics [88,89].

### 7.3. Efficiency and Sensitivity

**Figure 7:**
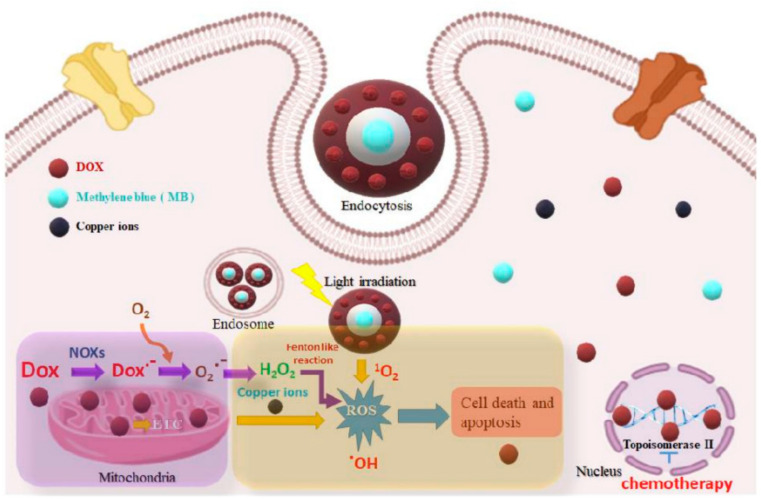
Diagram illustrating the likely working processes of the core-shell nanocomposites for photodynamic, chemodynamic, and chemotherapeutic treatments. Reprinted with permission from Ref. [91] https://doi.org/10.3390/ijms231911604.

Porous nanomaterials exhibit remarkable efficiency and sensitivity in biomedical applications, owing to their high surface area, tunable pore structures, and functionalization capabilities. For instance, nanoporous gold nanoparticles offer exceptional photothermal conversion efficiency, enabling efficient cancer cell ablation in photothermal therapy [90]. Figure 7 illustrates the operational mechanisms of core-shell nanocomposites in various therapeutic applications, including photodynamic, chemodynamic, and chemotherapeutic treatments. The diagram highlights how these nanocomposites, with a core-shell structure, enhance therapeutic efficacy through distinct processes. In photodynamic therapy (PDT), the core-shell nanocomposites absorb light and generate reactive oxygen species to destroy cancer cells. For chemodynamic therapy (CDT), they produce reactive oxygen species in response to specific triggers to induce oxidative stress and cell death. In chemotherapeutic applications, the nanocomposites facilitate targeted drug delivery, releasing therapeutic agents directly at the site of interest. The figure effectively demonstrates how the core-shell architecture optimizes treatment outcomes by integrating multiple therapeutic techniques into a single nanocarrier system [91]. Similarly, dendritic mesoporous silica nanoparticles (DMSNs) exhibit a high ability to load drugs and sustained release kinetics, increasing the effectiveness of anticancer drug treatments [92]. Furthermore, the unique physicochemical properties of porous nanomaterials contribute to their exceptional sensitivity in biosensing and molecular imaging applications.

### 7.4. Targeting and Specificity

Porous nanomaterials offer precise targeting and specificity in biomedical applications, enabling selective accumulation at disease sites while minimizing off-target effects. Surface modification of these materials with targeting ligands or antibodies enhances their affinity towards specific biomarkers or cellular receptors, facilitating targeted drug delivery or imaging [76,93,94]. Furthermore, the modular design of porous nanomaterials allows for the incorporation of multiple targeting moieties, enabling multimodal targeting strategies for enhanced specificity and therapeutic efficacy.

## 8. Challenges and Limitations

### 8.1. Biocompatibility and Toxicity

**Figure 8:**
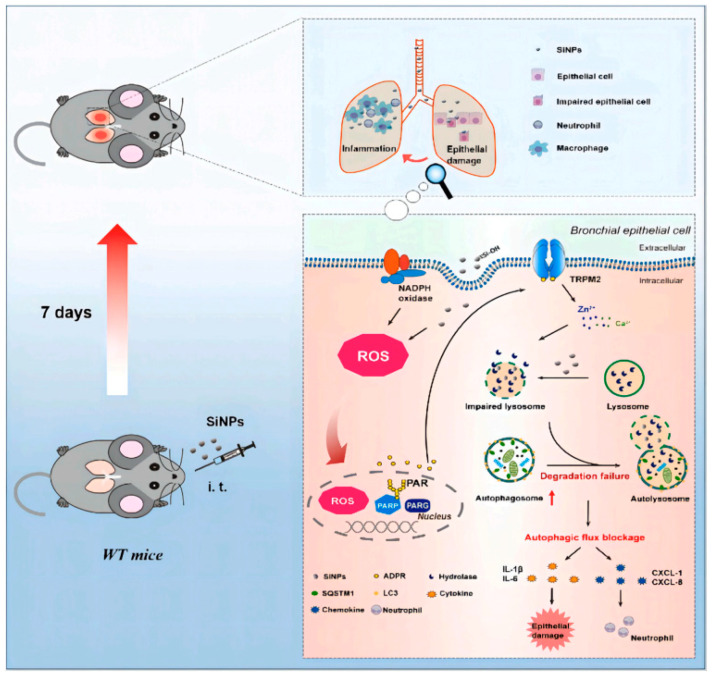
Schematic depicting the signaling pathway ROS/PARP/TRPM2 that causes SiNPs-induced lung inflammation in mice. Reprinted with permission from Ref. [98] https://doi.org/10.1186/s12989-020-00353-3.

One of the main obstacles to the use of porous nanomaterials for biomedical purposes is ensuring their biocompatibility and minimizing potential toxicity issues. While many porous materials exhibit excellent biocompatibility, certain factors like surface functionalization, particle size, and degradation products can affect their interactions with biological systems [95,96,97]. Figure 8 illustrates the signaling pathway involving ROS/PARP/TRPM2 that mediates lung inflammation induced by silicon nanoparticles (SiNPs) in mice. Upon intratracheal exposure to SiNPs, bronchial epithelial cells internalize these particles, leading to the generation of reactive oxygen species (ROS). These ROS trigger the activation of poly (ADP-ribose) polymerase (PARP), which synthesizes ADP-ribose (ADPR) in the nucleus. The increase in ADPR activates the TRPM2 ion channel, resulting in elevated intracellular levels of zinc and calcium ions. This ion accumulation impairs lysosomal function, which disrupts the production and release of key inflammatory cytokines and chemokines, such as IL-1β, IL-6, CXCL-1, and CXCL-8, ultimately contributing to the inflammatory response [98]. Understanding the mechanisms underlying nanoparticle-cell interactions and conducting comprehensive biocompatibility assessments are essential for the safe translation of porous nanomaterials into clinical applications.

### 8.2. Stability and Degradation

Another significant limitation of porous nanomaterials in biomedical applications is their stability and potential degradation over time. Factors like pH, temperature, and enzymatic activity in biological environments can affect the structural integrity and functionality of porous materials [99,100,101]. Developing strategies to enhance the stability and prolong the lifespan of porous nanomaterials is crucial for ensuring their efficacy and safety in clinical settings.

### 8.3. Scale-Up and Manufacturing

Upscaling and manufacturing porous nanomaterials for biomedical applications pose significant challenges due to the complexity of synthesis methods and the need for stringent quality control [102,103,104]. Achieving resproducible synthesis at large scales while maintaining the desired physicochemical properties and performance characteristics is essential for clinical applications. Additionally, cost-effective and scalable manufacturing processes are required to facilitate the widespread adoption of porous nanomaterials in healthcare.

### 8.4. Regulatory and Ethical Issues

Navigating regulatory pathways and addressing ethical considerations are essential steps in the development and translation of porous nanomaterials for biomedical applications [105,106,107]. Regulatory agencies require scomprehensive safety and efficacy data to approve the clinical use of porous materials, requiring rigorous preclinical testing and adherence to regulatory guidelines. Moreover, ethical concerns regarding patient privacy, informed consent, and potential misuse of nanotechnology must be addressed to ensure the responsible and ethical uset of porous nanomaterials in healthcare settings. The clinical application of nanoporous materials in biomedicine is a promising frontier, offering innovative solutions for drug delivery, tissue engineering, and diagnostic applications. However, their development and clinical translation are subject to stringent regulatory and ethical scrutiny. This section delves into the regulatory pathways and ethical considerations that are critical to the responsible and safe deployment of nanoporous materials in healthcare.

#### 8.4.1. Regulatory Pathways for Clinical Approval

Regulatory agencies such as the U.S. Food and Drug Administration (FDA), the European Medicines Agency (EMA), among others, play pivotal roles in ensuring that new medical technologies, including nanoporous materials, meet safety and efficacy standards before they can be marketed.

a. Preclinical testing

Before clinical trials can begin, nanoporous materials must undergo comprehensive preclinical testing. These tests assess biocompatibility, toxicity, pharmacokinetics, and pharmacodynamics, often using in vitro assays and animal models. For instance, the ISO 10993 series provides guidelines for the biological evaluation of medical devices, including those containing nanomaterials (ISO, 2018) [105].

b. Clinical trials

Following successful preclinical studies, nanoporous materials must pass through several phases of clinical trials:

  - Phase I focuses on safety and involves a small number of healthy volunteers or patients;
  - Phase II assesses efficacy and side effects in a larger group:
  - Phase III involves large-scale testing to confirm effectiveness, monitor side effects, and compare with commonly used treatments.

The FDA’s 21 CFR Part 312 regulations outline the investigational new drug (IND) application process, which must be submitted and approved before commencing clinical trials (FDA, 2020).

c. Market approval and post-market surveillance

Once clinical trials demonstrate safety and efficacy, the developer must submit a new drug application (NDA) or biologics license application (BLA) to the FDA, or a similar dossier to the EMA. Approval is granted based on a thorough review of the clinical data. Post-market surveillance, including adverse event reporting and long-term safety studies, is critical to monitor the ongoing safety and efficacy of the products (FDA, 2020; EMA, 2014).

#### 8.4.2. Regulatory Challenges Specific to Nanoporous Materials

Nanoporous materials present unique regulatory challenges due to their novel properties, such as their high surface area and unique interactions with biological systems.

a. Characterization and standardization

Accurate characterization of nanoporous materials, including size, shape, surface chemistry, and porosity, is crucial for regulatory approval. However, the lack of standardized methods and terminology can complicate the evaluation process. Efforts by organizations like the International Organization for Standardization (ISO) and the National Institute of Standards and Technology (NIST) are ongoing to establish standardized testing protocols (NIST, 2020).

b. Risk assessment

Nanomaterials can exhibit unpredictable toxicological profiles due to their small size and high reactivity. Regulators require detailed risk assessments covering potential toxicities, including oxidative stress, inflammatory responses, and genotoxicity. Special attention is given to chronic exposure and the potential for bioaccumulation [105].

c. Manufacturing and quality control

The production of nanoporous materials must adhere to good manufacturing practices (GMP). Consistency in material properties between batches is critical, as minor variations can lead to significant differences in biological responses. Regulatory agencies rigorously scrutinize manufacturing processes and quality control measures to ensure product safety and effectiveness (EMA, 2019).

#### 8.4.3. Ethical Considerations

The use of nanoporous materials in healthcare also raises significant ethical issues, requiring a thoughtful approach to their development and deployment.

a. Informed consent

Patients must be fully informed about the risks and benefits of treatments involving nanoporous materials. This is particularly challenging given the complex nature of nanotechnology, which may be difficult for patients to understand. Ensuring that patients have a clear understanding and can make an informed decision is a key ethical obligation [106].

b. Privacy and data protection

The use of nanoporous materials in diagnostics and drug delivery may involve the collection of sensitive biological data. Protecting patient privacy and ensuring that data is used ethically are paramount. Compliance with regulations such as the Health Insurance Portability and Accountability Act (HIPAA) in the United States and the General Data Protection Regulation (GDPR) in the European Union is essential (HIPAA, 1996; GDPR, 2016).

c. Equity and access

Ensuring equitable access to advanced medical treatments is a significant ethical concern. The high cost of developing nanoporous materials and associated therapies can limit their availability, potentially exacerbating healthcare inequalities. Ethical considerations demand that efforts be made to make these treatments accessible to a broad patient population.

d. Environmental and long-term impact

The long-term environmental impact of nanoporous materials, particularly in terms of biodegradability and potential toxicity, must be considered. The ethical development of these materials includes assessing their lifecycle impact and ensuring sustainable practices in their production and disposal [107].

#### 8.4.4. Regulatory and Ethical Framework Development

The rapid advancement of nanotechnology requires the continuous evolution of regulatory frameworks and ethical guidelines. This includes:

- Interdisciplinary collaboration: involving experts from toxicology, material science, medicine, ethics, and law to develop comprehensive guidelines;
- Public engagement: engaging the public in discussions about the benefits and risks of nanoporous materials to build trust and support informed decision-making;
- International cooperation: harmonizing regulations across countries to facilitate global access to safe and effective nanotechnology-based treatments.

The development of nanoporous materials for biomedical applications holds great promise but must be navigated with care through a complex landscape of regulatory and ethical challenges. Comprehensive preclinical testing, rigorous clinical trials, and adherence to ethical principles are crucial to ensuring the safe and equitable use of these advanced materials. As the field continues to evolve, ongoing dialogue among scientists, regulators, ethicists, and the public will be essential to harnessing the full potential of nanoporous materials while safeguarding public health and trust.

## 9. Future Perspectives

### 9.1. Emerging Trends in Nanoporous Materials

The field of nanoporous materials is witnessing rapid advancements, driven by emerging trends that hold promise for various biomedical applications. One such trend is the development of stimuli-responsive porous nanomaterials that can undergo controlled structural alterations in reaction to outside stimuli like light, pH, or temperature [108,109,110]. These dynamic materials offer opportunities for on-demand drug release, targeted therapy, and real-time monitoring of biological processes. Additionally, the integration of artificial intelligence and machine learning into nanoporous material construction and characterization processes is facilitating the discovery of novel materials with enhanced properties and functionalities.

### 9.2. Innovations in Synthesis and Functionalization

Innovations in synthesis and functionalization techniques are driving the development of advanced nanoporous materials with tailored properties and enhanced performance characteristics. Recent breakthroughs in bottom-up synthesis methods, like atomic layer deposition and chemical vapor deposition, enable precise control over pore size, shape, and surface chemistry [111,112,113]. Furthermore, the advent of bio-inspired and bio-assisted synthesis methods allows for the fabrication of porous materials with biomimetic structures and functionalities, opening new avenues for tissue engineering, regenerative medicine, and drug delivery applications.

### 9.3. Potential Breakthroughs in Imaging and Diagnostics

The future of biomedical imaging and diagnostics holds exciting possibilities with the advent of novel nanoporous material-based platforms. Advances in nanoparticle engineering and surface functionalization strategies are enabling the development of highly sensitive and specific imaging probes for early disease detection and personalized medicine [114,115,116]. Moreover, the integration of multimodal imaging techniques and theranostic functionalities into single nanoparticle systems hsolds promise for real-time monitoring of therapeutic responses and disease progression.

### 9.4. Interdisciplinary Approaches

The convergence of diverse scientific disciplines, including materials science, chemistry, biology, and engineering, is driving interdisciplinary research efforts towards the development of innovative nanoporous material-based solutions. Collaborations between scientists, engineers, and clinicians are essential for addressing complex healthcare challenges and translating nanoporous materials from bench to bedsside [117,118,119]. Furthermore, interdisciplinary training programs and educational initiatives are fostering the next generation of researchers equipped with the knowledge and skills to tackle multifaceted healthcare problems.

### 9.5. Advancements in Nanoporous Materials

1. Enhanced surface area and porosity: Nanoporous materials exhibit significantly larger surface areas compared to other nanomaterials, facilitating improved interaction with biological molecules. This property enhances applications in drug delivery, catalysis, and biosensing. For instance, mesoporous silica nanoparticles can be designed with specific pore sizes for controlled drug release, an advancement over non-porous nanoparticles that lack such precise control.
2. Controlled drug release: The capability of nanoporous materials to provide controlled and sustained drug release is a major advancement. This allows for the precise tuning of release rates, which is crucial for managing chronic conditions. For example, mesoporous silica nanoparticles can release drugs over extended periods of time, reducing the need for frequent dosing.
3. Functionalization flexibility: The surface chemistry of nanoporous materials can be modified to enhance biocompatibility and target specificity, surpassing the functionalization capabilities of some other nanomaterials. For instance, surface modifications on mesoporous silica can improve compatibility with biological systems or target specific cells, offering a level of customization not easily achievable with other materials.
4. Biocompatibility and safety: Certain nanoporous materials, such as silica, are highly biocompatible and crucial for biomedical applications. They can be engineered to degrade safely within the body, minimizing potential toxicity—a common issue with some other nanomaterials like certain metal oxides.

### 9.6. Disadvantages and Considerations

1. Complex synthesis and scalability: The complex synthesis processes of nanoporous materials pose challenges for scalability, potentially limiting their widespread use compared to other nanomaterials that are easier to produce at scale.
2. Potential for unintended bio-interactions: The high surface area and porosity of these materials, while advantageous, may lead to unintended interactions with biological molecules, potentially causing off-target effects. This includes the possible activation of the immune system or unwanted protein interactions.
3. Regulatory and ethical challenges: Nanoporous materials face stringent regulatory and ethical scrutiny due to their novel properties and potential risks. Regulatory approval can be challenging, impacting their clinical application.
4. Potential for accumulation and toxicity: The potential for accumulation and toxicity is a concern, particularly for materials that are not easily degraded or cleared from the body, posing long-term safety and environmental risks.

So, while nanoporous materials present significant advancements, particularly in controlled drug release and functionalization, these benefits must be weighed against potential disadvantages, including complex synthesis and potential toxicity. Further research and careful consideration are needed to ensure safe and effective clinical applications.

## 10. Conclusions

### 10.1. Summary of Key Findings

In summary, this review highlights the significant contributions of nanoporous materials to biomedical imaging, diagnostics, and therapeutic applications. Through a comprehensive analysis of current research trends and advancements, several key findings have emerged. Nanoporous materials offer versatile platforms for targeted drug delivery, controlled release, and image-guided therapies, owing to their tunable pore structures, high surface areas, and functionalization capabilities [120,121,122]. Furthermore, the integration of multimodal imaging techniques and theranostic functionalities into nanoposrous materials holds promise for personalized medicine and precision healthcare.

### 10.2. Implications for Biomedical Research

The implications of nanoporous materials for biomedical research are profound, offering transformative opportunities to address unmet clinical needs and improve patient outcomes. By harnessing the unique properties of nanoporous materials, researchers can develop innovative solutions for targeted drug delivery, non-invasive imaging, and early disease detection [88,123,124]. Moreover, the versatility and scalability of nanoporous material synthesis techniques enable the rapid translation of research findings from the laboratory to clinical practice, paving the way for the development of next-generation medical technologies.

#### Nanoporous Materials in Clinical Practice: Rapid Transition from Lab to Clinic

Nanoporous materials have demonstrated significant potential for clinical applications due to their unique properties, including high surface area, tunable pore sizes, and biocompatibility. These characteristics make them suitable for various biomedical applications, from drug delivery systems to diagnostic tools. The rapid transition from laboratory research to clinical practice is facilitated by these properties, along with advances in scalable synthesis methods and comprehensive preclinical studies.

1. Drug delivery systems

One of the most well-explored applications of nanoporous materials is in the design of drug delivery systems. For example, mesoporous silica nanoparticles (MSNs) have been extensively studied for their ability to deliver chemotherapeutic agents to cancer cells. These nanoparticles can be functionalized with targeting ligands, such as folic acid, which binds specifically to cancer cells overexpressing folate receptors. This targeting capability enhances the efficacy of the drug while minimizing side effects. A notable study demonstrated the use of MSNs to deliver doxorubicin, a common chemotherapy drug, directly to breast cancer cells, significantly improving therapeutic outcomes compared to conventional methods.

2. Imaging and diagnostics

Nanoporous materials are also pivotal in enhancing imaging techniques. Gold nanoparticles, for instance, have been developed to improve the contrast in computed tomography (CT) scans. These nanoparticles can be designed to accumulate in specific tissues, providing enhanced imaging of areas such as tumors or vascular anomalies. A research team successfully used gold nanoporous shells to increase the imaging contrast for CT scans, enabling clearer visualization of tumors. This capability is crucial for early and accurate diagnosis, leading to better treatment planning and outcomes.

3. Biosensing and early disease detection

The high sensitivity of nanoporous materials makes them ideal for biosensing applications, which are essential for early disease detection. For example, porous silicon-based sensors have been developed for the detection of biomarkers associated with diseases such as cancer and diabetes. These sensors can detect very low concentrations of biomarkers, providing early indications of disease. In one study, porous silicon biosensors were used to detect the prostate-specific antigen (PSA), a marker for prostate cancer, at nanomolar concentrations. Such early detection can lead to earlier intervention and an improved prognosis.

4. Scaffold materials in tissue engineering

Nanoporous materials are also employed as scaffolds in tissue engineering, providing a framework that supports cell growth and differentiation. For instance, nanoporous hydroxyapatite has been used as a scaffold material for bone tissue regeneration. The porosity and bioactivity of these materials support the adhesion and proliferation of osteoblasts, which are essential for effective bone regeneration. A study demonstrated the efficacy of nanoporous hydroxyapatite in promoting the growth of new bone tissue, suggesting its potential for use in clinical bone repair procedures.

5. Translational Research and Clinical Trials

The rapid progression of nanoporous materials from laboratory research to clinical trials is supported by advancements in their synthesis and functionalization. The ability to produce these materials in a scalable and reproducible manner is critical for their translation into clinical settings. Moreover, the integration of nanoporous materials with existing medical technologies, such as drug delivery devices and diagnostic tools, facilitates their adoption in clinical practice.

Ultimately, the unique properties of nanoporous materials, combined with advancements in their synthesis and functionalization, have enabled a swift transition from research to clinical applications. These materials offer significant benefits in drug delivery, imaging, biosensing, and tissue engineering, providing new solutions to longstanding clinical challenges.

### 10.3. Final Remarks

In conclusion, nanoporous materials represent a paradigm shift in biomedical research and hold immense potential for revolutionizing healthcare diagnostics and treatment strategies. As we continue to unravel the complexities of nanoporous materials and explore their diverse applications, interdisciplinary collaborations and translational efforts will be instrumental in unlocking their full clinical utility [125,126,127]. By leveraging the combined knowledge of researchers, engineers, healthcare professionals, and regulatory bodies, we can accelerate the development and implementation of technologies based on nanoporous materials. This collaboration aims to tackle global health issues and significantly enhance the quality of life for millions of people worldwide.
